# The causal relationship between 5 serum lipid parameters and diabetic nephropathy: a Mendelian randomization study

**DOI:** 10.3389/fendo.2024.1358358

**Published:** 2024-05-28

**Authors:** Hongzhou Liu, Xinxia Yao, Linlin Wang, Jia Liu, Xiaojing Li, Xiaomin Fu, Jing Liu, Song Dong, Yuhan Wang

**Affiliations:** ^1^ Department of Endocrinology, Aerospace Center Hospital, Beijing, China; ^2^ Department of Endocrinology, First Hospital of Handan City, Handan, Hebei, China; ^3^ Medical-Education Collaboration and Medical Education Research Center, Hebei Medical University, Shijiazhuang, Hebei, China; ^4^ Clinics of Cadre, Department of Outpatient, The First Medical Center, Chinese People's Liberation Army (PLA) General Hospital, Beijing, China; ^5^ Department of Endocrinology, Beijing Friendship Hospital, Capital Medical University, Beijing, China

**Keywords:** Mendelian randomization, serum, lipid, diabetic nephropathy, instrumental variable

## Abstract

**Background:**

Serum lipids were found to be correlated with chronic kidney disease and cardiovascular disease. Here, we aimed to research the potential causal associations between five serum lipid parameters and the risk of diabetic nephropathy using several Mendelian Randomization methods.

**Methods:**

Genetic data was obtained from the UK Biobank datasets. Causal effects were estimated using multiple MR methods. Heterogeneity and pleiotropy tests were performed.

**Results:**

MR analysis revealed that HDL-C and TG exhibited causal associations with diabetic nephropathy (*P*<0.05). Similar trends were not observed for other lipid parameters.

**Conclusions:**

Our research has suggested links between HDL-C, TG and diabetic nephropathy. The findings could contribute to further elucidation of the disease etiology.

**Strengths and limitations of this study:**

This article only uses Mendel randomization method to analyze the relationship between blood lipids and diabetes nephropathy, which is more convincing when combined with population data.

## 1 Introduction

Diabetic nephropathy is a progressive kidney disease that arises as a severe complication of diabetes mellitus (1). It is characterized by gradual damage to the kidneys’ delicate filtering units, known as nephrons, due to high levels of blood sugar over an extended period of time. This condition is a significant concern for individuals with either type 1 or type 2 diabetes (2). Timely detection and management of diabetic nephropathy are crucial to slow its progression and reduce the risk of severe complications.

Previous studies have delved into the correlation between several serum lipid parameters and various diseases. For example, serum lipids were found to be correlated with chronic kidney disease and cardiovascular disease (3, 4). Previous studies have revealed the potential of dyslipidemia to trigger renal vascular atherosclerosis, glomerulosclerosis, and tubulointerstitial damage, leading to deterioration of renal function (5, 6). Some studies have unveiled links between elevated levels of blood lipids like total cholesterol (TC), low-density lipoprotein cholesterol (LDL-c), triglycerides (TG), and a reduced level of blood high-density lipoprotein cholesterol (HDL-c), and an elevated risk of incident diabetic nephropathy. Nevertheless, the evidence stemming from these population-based studies has exhibited inconsistencies and faced challenges in establishing a definitive causal relationship (2, 7, 8).

Mendelian Randomization studies have gained increasing popularity for assessing causal relationships among risk factors. The availability of extensive GWAS meta-analyses has facilitated the application of MR to investigate whether risk factors with a genetic basis exert causal effects on health outcomes (9). Yet, it’s important to acknowledge that the estimation of causal effects through MR can be influenced by pleiotropy, where genetic variants are linked to intermediate traits that contribute to the outcome through alternative pathways (10). To address this, specialized techniques like Egger approaches were devised to identify and address potential pleiotropic effects. By employing a comprehensive MR framework and leveraging data from extensive GWAS, our study aims to explore serum lipid parameters (HDL-C, LDL-C, total cholesterol, triglycerides, and lipoprotein-a)and their relationships with diabetic nephropathy, thereby offering support for a plausible causal relationship (11).

## 2 Methods

### 2.1 Data source

Genetic data for this Mendelian Randomization analysis were sourced from the UK Biobank dataset, accessed through (https://gwas.mrcieu.ac.uk/datasets/). Keywords used for searching are “total cholesterol”, “triglyceride”, “LDL”, “HDL”, “lipoprotein a”. Specific datasets for each blood lipid component were as follows:

1. Total Cholesterol (TC): Met-D-Total_C (2020, UK Biobank). The dataset contained European males and females, and the sample size was 115,078.
2. Triglycerides (TG): ieu-b-111 (2020, UK Biobank). The dataset contained European males and females, and the sample size was 441,016.
3. LDL-C: ieu-b-110 (2020, UK Biobank). The dataset contained European males and females, and the sample size was 440,546.
4. HDL-C: ieu-b-109 (2020, UK Biobank). The dataset contained European males and females, and the sample size was 403,943.
5. Lipoprotein(a): ieu-b-107 (2020, UK Biobank). The dataset contained European males and females, and the sample size was 393,193.

### 2.2 Study design

1. Genetic Instrument Selection: Single nucleotide polymorphisms (SNPs) associated with each blood lipid component were selected as instrumental variables from the respective datasets. SNPs were chosen based on stringent criteria, including genome-wide significance and relevance to the specific lipid component.
2. Exposure and Outcome Variables: The serum lipid parameters (exposure) were investigated for their potential causal effects on diabetic nephropathy (outcome).
3. Assumptions: Three assumptions were examined to establish the suitability of SNPs as genetic instrumental variables for unbiasedly estimating the causal impact. These assumptions encompass: 1) the genetic instrumental variables should demonstrate consistent and robust associations with the exposure; 2) these variables should remain unrelated to confounding factors; 3) the genetic instrumental variables should not exhibit a direct connection to the outcome; rather, their influence on the outcome should be mediated solely through their association with the exposure (12).

### 2.3 Statistical analysis

The SNP effect on nephropathy was assessed using six methods: MR Egger, Weighted median, Inverse variance weighted (IVW), Maximum likelihood, Penalized weighted median and MR-RAPS. Among these methodologies, we employed IVW as the primary statistical technique in our investigation, given that it could provide the most precise estimates of causal effects. Beta coefficients (β) and odds ratios (OR) were calculated for each blood lipid parameter. Heterogeneity tests and tests for horizontal pleiotropy were performed to evaluate potential biases. An observed p-value below 0.05 indicated the existence of pleiotropy. In instances where substantial heterogeneity or horizontal pleiotropy was identified, MR-PRESSO approach was employed to identify and exclude outlier SNPs. To enhance the robustness of our analysis, we performed a sensitivity assessment employing a “leave-one-out” approach. This involved iteratively excluding one variant at a time and reperforming the MR analyses for each approach.

#### 2.3.1 MR egger

The inclusion of a non-zero y-intercept is permissible to detect deviations from conventional MR assumptions. The presence of a statistically significant intercept serves as an indicator for potential unmeasured pleiotropic influences introduced by the genetic instrument. However, a notable drawback of this approach is a substantial reduction in statistical power. MR Egger might be influenced by substantial effects from unusual genetic factors, leading to imprecise estimations. Nevertheless, the MR-Egger test has the capacity to yield unbiased estimates (13).

#### 2.3.2 Weighted median

The weighted median estimator has the capability to offer a reliable causal estimate, even in scenarios where as much as 50% of the information from instrumental variables (IVs) is non-operational (14).

#### 2.3.3 IVW

This method is rooted in the principle of exploiting genetic variants’ random allocation during meiosis, thereby mimicking a randomized controlled trial to investigate causal associations in observational data. The IVW method offers a powerful tool to address causal inquiries and illuminate relationships between variables of interest in a robust and statistically sound manner within the framework of MR (11, 15).

#### 2.3.4 Maximum likelihood

By maximizing the likelihood function, this method provides parameter estimates that optimize the fit between the observed data and the underlying causal model. The ML approach is particularly valuable when addressing potential violations of key assumptions, such as horizontal pleiotropy or heterogeneous genetic effects, as it can yield more accurate and precise estimates while acknowledging the complexity of real-world genetic relationships (11).

#### 2.3.5 Penalized weighted median

A modification of the traditional weighted median approach, PWM introduces a penalty term that downweighs the contribution of individual genetic variants based on their estimated pleiotropic effects. This method allows for a nuanced exploration of causal relationships, taking into account potential biases and uncertainties inherent in genetic instrument selection (16).

#### 2.3.6 MR-RAPS

MR-RAPS utilizes a series of weighted instrumental variables to estimate the causal effect while minimizing the influence of outliers and pleiotropic variants. This method offers a robust way to account for complex genetic relationships and potential confounding factors, ultimately yielding more reliable estimates of causal relationships between exposures and outcomes (17).

#### 2.3.7 MR-PRESSO

The MR-PRESSO technique was utilized for identifying and addressing horizontal pleiotropic outliers in MR analysis (18).

### 2.4 Software

Data processing and analysis were performed using R version 4.3.0, along with Storm Statistical Platform (www.medsta.cn/software) and Mendelian Randomization package (19).

## 3 Results

### 3.1 Association between serum lipid parameters and diabetic nephropathy

**Figure 1:**
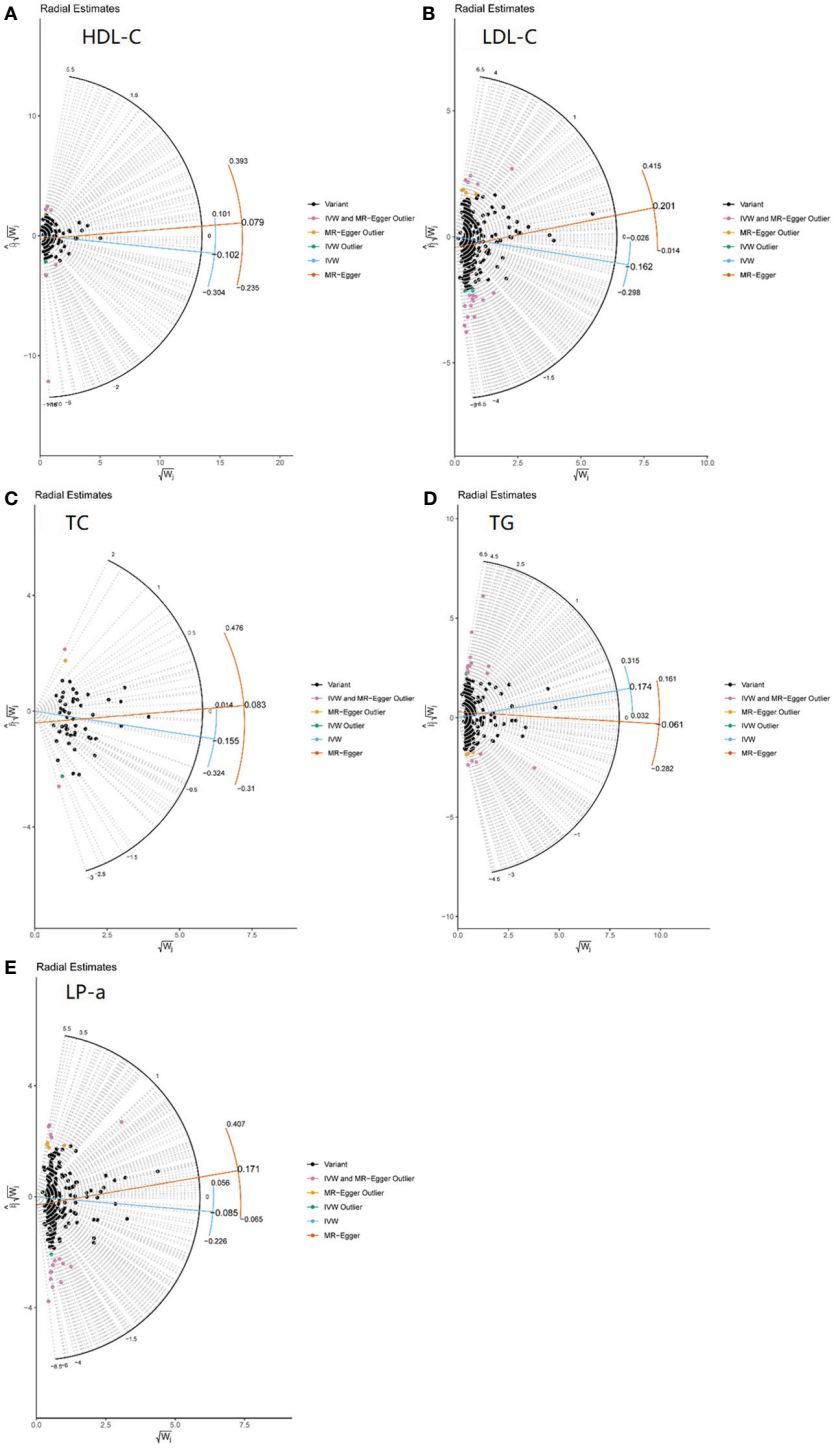
Radial MR plot of 5 lipid parameters. **(A)** HDL-C. **(B)** LDL-C. **(C)** TC. **(D)** TG. **(E)** LP-a.

The associations between serum lipid parameters and diabetic nephropathy are shown in **Table 1**. RadialMR graphs of each lipid parameter were shown in **Figure 1**.

**Table 1:** β and OR values for 5 blood lipid parameters using 6 calculation methods.

Blood lipid		HDL-C	LDL-C	TC	TG	Lp_a
SNP		304	151	55	270	253
	β	0.13	0.07	-0.03	0.01	0.11
MR Egger	OR (95% *CI*)	1.14 (0.93-1.39)	1.07(0.80-1.43)	0.98(0.69-1.35)	1.01(0.82-1.24)	1.12(0.89-1.39)
	P	0.21	0.65	0.86	0.91	0.33
	β	0.02	-0.03	-0.05	0.10	0.08
Weighted median	OR (95% *CI*)	1.02 (0.83-1.25)	0.97(0.77-1.23)	0.95(0.73-1.23)	1.01(0.88-1.40)	1.08(0.87-1.34)
	P	0.86	0.79	0.70	0.40	0.48
	β	-0.16	-0.10	-0.15	0.17	-0.09
IVW	OR (95% *CI*)	0.85 (0.74-0.97)	0.90(0.74-1.10)	0.86(0.72-1.02)	1.19(1.03-1.37)	0.92(0.80-1.06)
	P	0.02	0.32	0.08	0.02	0.24
	β	-0.16	-0.10	-0.16	0.18	-0.09
Maximum likelihood	OR (95% *CI*)	0.85 (0.75-0.96)	0.90(0.78-1.04)	0.86(0.72-1,02)	1.19(1.05-1.34)	0.92(0.81-1.05)
	P	0.01	0.17	0.07	0.01	0.20
	β	0.02	-0.03	-0.05	0.10	0.08
Penalised weighted median	OR (95% *CI*)	1.02 (0.83-1.25)	0.97(0.77-1.21)	0.95(0.73-1.25)	1.19(1.05-1.35)	1.08(0.87-1.34)
	P	0.85	0.79	0.72	0.40	0.48
	β	-0.15	-0.04	-0.14	0.15	-0.07
Mr-raps	OR (95% *CI*)	0.86(0.75-0.99)	0.97(0.83-1.12)	0.87(0.73-1,04)	1.16(1.02-1.33)	0.94(0.82-1.07)
	P	0.04	0.64	0.11	0.02	0.33

**Figure 2:**
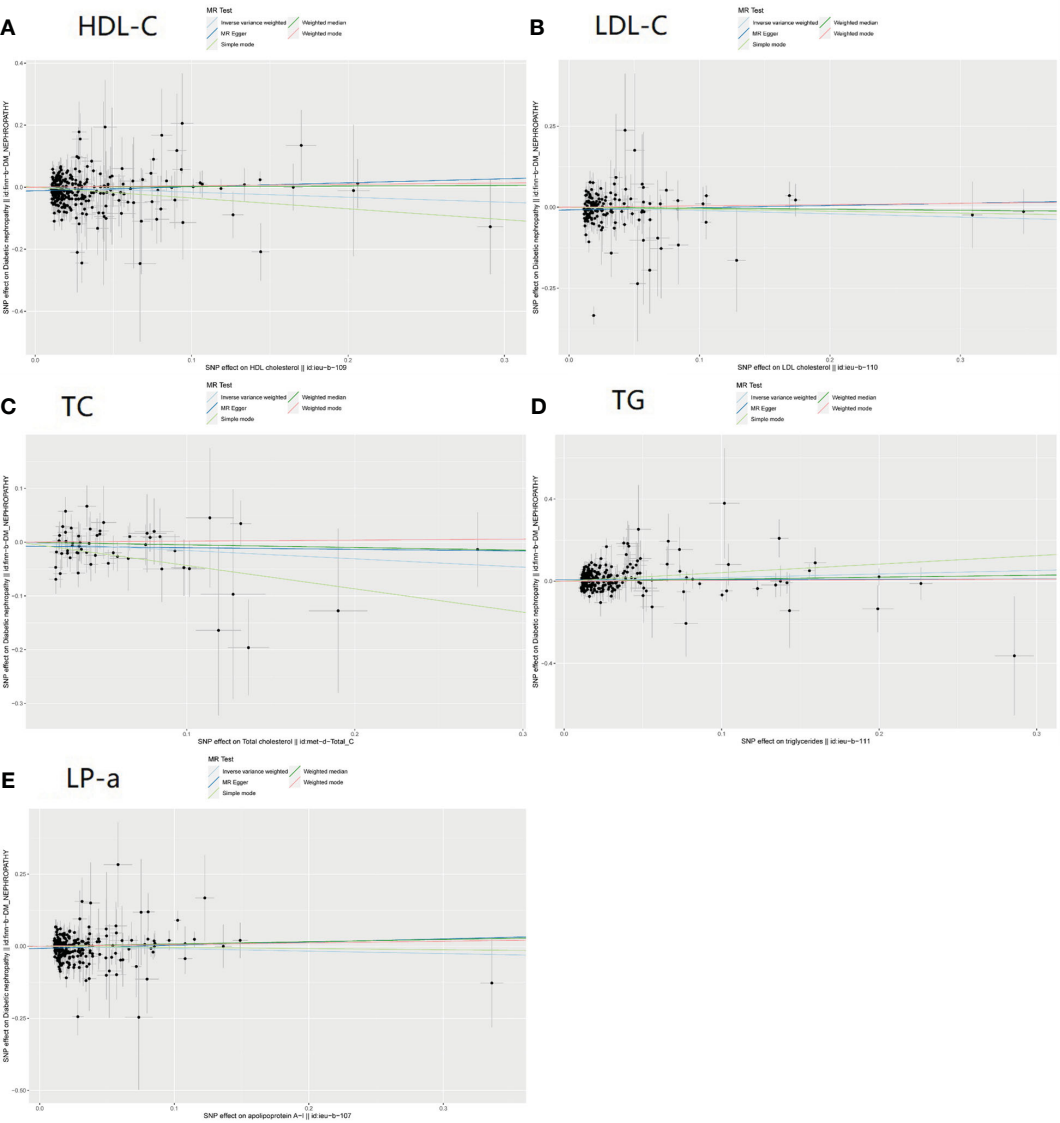
Scatter plot of 5 blood lipid parameters. **(A)** HDL-C. **(B)**B LDL-C. **(C)** TC. **(D)** TG. **(E)** LP-a.

1) For HDL-C, we observed the following associations using different MR methods (**Figure 2A**)

SNP=304

- MR Egger: The beta coefficient (β) was 0.13. The odds ratio (OR) based on the MR Egger method was 1.14 (95% confidence interval [CI]: 0.93-1.39), with a p-value of 0.21.
- Weighted Median: The OR was 1.02 (95% CI: 0.83-1.25), yielding a p-value of 0.86.
- IVW (Inverse Variance Weighting): The OR was 0.85 (95% CI: 0.74-0.97), with a p-value of 0.02.
- Maximum Likelihood: The OR was 0.85 (95% CI: 0.75-0.96), with a p-value of 0.01.
- Penalized Weighted Median: The OR was 1.02 (95% CI: 0.83-1.25), and the p-value was 0.85.
- MR-RAPS (Mendelian Randomization with Robust Adjusted Profile Score): The OR was 0.86 (95% CI: 0.75-0.99), yielding a p-value of 0.04.

2) For LDL-C, the results are as follows (**Figure 2B**):

SNP=151

- MR Egger: β was 0.07, indicating a positive association. The OR was 1.07 (95% CI: 0.80-1.43), with a p-value of 0.65.
- Weighted Median: The OR was 0.97 (95% CI: 0.77-1.23), with a p-value of 0.79.
- IVW: The OR was 0.90 (95% CI: 0.74-1.10), yielding a p-value of 0.32.
- Maximum Likelihood: The OR was 0.90 (95% CI: 0.78-1.04), and the p-value was 0.17.
- Penalized Weighted Median: The OR was 0.97 (95% CI: 0.77-1.21), with a p-value of 0.64.
- MR-RAPS: The OR was 0.97 (95% CI: 0.83-1.12), and the p-value was 0.02.

3) Total Cholesterol (**Figure 2C**)

SNP=55

- MR Egger Analysis: The beta coefficient (β) for TC was -0.03. The odds ratio (OR) from the MR Egger analysis was 0.98 (95% confidence interval [CI]: 0.69-1.35), and the associated p-value was 0.86.
- Weighted Median Method: The OR for TC was 0.95 (95% CI: 0.73-1.23), resulting in a p-value of 0.70.
- IVW Method: We observed an OR of 0.86 (95% CI: 0.72-1.02) for TC, with a p-value of 0.08.
- Maximum Likelihood Analysis: The OR calculated using the Maximum Likelihood method was 0.86 (95% CI: 0.72-1.02), and the corresponding p-value was 0.07.
- Penalized Weighted Median Method: The OR for TC was 0.95 (95% CI: 0.73-1.25), yielding a p-value of 0.72.
- MR-RAPS Analysis: The OR derived from the MR-RAPS method was 0.87 (95% CI: 0.73-1.04), and the associated p-value was 0.11.

4) Triglycerides (**Figure 2D**)

SNP=270

Our analysis of genetic variants and triglyceride (TG) levels yielded the following results:

- MR Egger Analysis: The beta coefficient (β) for TG was 0.01. The OR calculated using the MR Egger method was 1.01 (95% CI: 0.82-1.24), and the associated p-value was 0.91.
- Weighted Median Method: The OR for TG was 1.01 (95% CI: 0.88-1.40), resulting in a p-value of 0.40.
- IVW Method: We observed an OR of 1.19 (95% CI: 1.03-1.37) for TG, with a p-value of 0.02.
- Maximum Likelihood Analysis: The OR obtained through the Maximum Likelihood method was 1.19 (95% CI: 1.05-1.34), and the corresponding p-value was 0.01.
- Penalized Weighted Median Method: The OR for TG was 1.19 (95% CI: 1.05-1.35), yielding a p-value of 0.40.
- MR-RAPS Analysis: The OR derived from the MR-RAPS method was 1.16 (95% CI: 1.02-1.33), and the associated p-value was 0.02.

5) Lipoprotein(a) (**Figure 2E**)

SNP=253

- MR Egger Analysis: The beta coefficient (β) for Lp_a was 0.11. The OR obtained from the MR Egger analysis was 1.12 (95% CI: 0.89-1.39), and the associated p-value was 0.33.
- Weighted Median Method: The OR for Lp_a was 1.08 (95% CI: 0.87-1.34), resulting in a p-value of 0.48.
- IVW Method: The OR derived from the IVW method was 0.92 (95% CI: 0.80-1.06), with a p-value of 0.24.
- Maximum Likelihood Analysis: The OR calculated using the Maximum Likelihood method was 0.92 (95% CI: 0.81-1.05), and the corresponding p-value was 0.20.
- Penalized Weighted Median Method: The OR for Lp_a was 1.08 (95% CI: 0.87-1.34), yielding a p-value of 0.48.
- MR-RAPS Analysis: The OR for Lp_a was 0.94 (95% CI: 0.82-1.07), and the associated p-value was 0.33.

### 3.2 Sensitivity analysis

We conducted the sensitivity analysis to assess the robustness of the results. The heterogeneity test and horizontal pleiotropy test were employed to evaluate potential biases (**Table 2**; **Supplementary Figures 1**–**3**), Results of MR-PRESSO were shown in **Table 3**.

- For HDL-C, LDL-C, and TG, the heterogeneity test yielded p-values less than 0.01, indicating significant heterogeneity.
- For LDL-C, the horizontal pleiotropy test had a p-value of 0.12.
- For HDL-C, the horizontal pleiotropy test had a p-value <0.01
- For TC, TG and lipoprotein(a), the horizontal pleiotropy test had p-values of 0.38, 0.04, and 0.03 respectively.

**Table 2:** Sensitivity analysis.

Blood lipid		HDL-C	LDL-C	TC	TG	Lp_a
Heterogeneity analysis	P	<0.01	<0.01	0.62	<0.01	0.051
Horizontal Pleiotropy test	P	<0.01	0.12	0.38	0.04	0.03

**Table 3:** Results of MR-PRESSO.

Blood lipid	Raw estimates				Outlier corrected estimates			
	N	OR	95% CI	P-value	N	OR	95% CI	P-value
HDL-C	304	0.85	0.74-0.97	0.02	302	0.86	0.75-0.98	0.02
LDL-C	151	0.91	0.74-1.10	0.33	150	0.95	0.82-1.09	0.45
TC	55	0.86	0.73-1.01	0.07	NA	NA	NA	NA
TG	270	1.19	1.04-1.37	0.01	267	1.20	1.06-1.37	0.01
Lp_a	253	0.92	0.81-1.06	0.27	252	0.94	0.82-1.07	0.36

NA, Not Available.

## 4 Discussion

Mendelian randomization (MR) studies have gained significant traction due to their inherent ability to mitigate confounding factors, setting them apart from observational studies. These studies have found extensive use in the estimation of causal or mediation effects that connect exposures to health outcomes. In this study, we employed Mendelian Randomization to investigate the potential causal relationship between five serum lipid parameters—HDL-C, LDL-C, total cholesterol, triglycerides, and lipoprotein(a)—and the risk of diabetic nephropathy. The results found a potential protective effect of high HDL-C levels and a possible adverse impact of elevated TG levels. While some methods revealed associations, others did not reach significance. Sensitivity analysis highlighted heterogeneity and potential pleiotropy, emphasizing the complexity of these relationships. This study contributes important insights into the intricate interplay between serum lipids and diabetic nephropathy.

The exploration of underlying biological mechanisms stands as a pivotal endeavor in comprehending the observed associations. Previous epidemiologic research has consistently identified noteworthy correlations between various blood serum parameters and diseases. Lanktree et al. found that elevated levels of HDL cholesterol due to genetic factors are causally linked to improved kidney function. However, changes in LDL cholesterol or triglyceride levels due to genetic alterations did not show any significant association with kidney function (20). Interestingly, Lee et al’s initial expectation was that elevated TG levels had association with higher risk of T2D. However, their findings revealed a counterintuitive outcome (21). Sigfrids’ research exhibited that remnant cholesterol concentration was indicative of both diabetic neuropathy progression and severe diabetic retinopathy (7). Kintu et al. found that there appears to be a causal link between genetically higher LDL-C and TC levels and elevated eGFR levels (22). A study found that elevated blood TG levels might be linked in a causal manner to an increased risk of CKD (23). At the same time, kidney function could also impact blood lipid levels (24).

HDL-C and its potential protective effects against diabetic nephropathy sheds light on multifaceted pathways: 1) Anti-Inflammatory: HDL-C’s anti-inflammatory attributes counteract endothelial dysfunction and suppress inflammatory cascades, potentially preserving renal function against diabetic nephropathy’s ravages.2) Antioxidant activity: amidst the oxidative stress prevailing in diabetic nephropathy, HDL-C’s antioxidant prowess offers a potential shield against cellular damage and fibrosis.3) Endothelial function: HDL-C’s role in upholding endothelial integrity is crucial in averting renal endothelial dysfunction’s contribution to diabetic nephropathy pathogenesis.4) Proteinuria regulation: HDL-C’s modulation of proteinuria through glomerular filtration barrier regulation contributes to the safeguarding of renal integrity. 5) Promotion of renal repair: HDL-C’s support in tissue regeneration and fibrosis reduction adds a layer of defense against irreversible kidney function decline (4, 25, 26).Triglyceride’s intricate role in diabetic nephropathy includes: 1) elevated levels of triglycerides are linked to microvascular dysfunction, which can lead to reduced blood flow and impaired nutrient delivery to the kidneys 2) Insulin resistance and inflammation: high triglyceride levels are often associated with insulin resistance and systemic inflammation; 3) oxidative stress and endothelial dysfunction: hypertriglyceridemia can induce oxidative stress and endothelial dysfunction; 4) elevated triglycerides may disrupt podocyte function, leading to increased glomerular permeability and proteinuria; 5) excessive triglyceride accumulation within renal cells can lead to lipo-toxicity. These hemodynamic changes may contribute to the progression of diabetic nephropathy (26, 27).

The results suggest potential associations between certain serum lipid parameters and diabetic nephropathy, but these associations should be viewed cautiously in light of the potential for bias and confounding. We acknowledge the potential for horizontal pleiotropy. Efforts were made to address this concern by utilizing various MR methods, each with its unique approach to handling pleiotropy. However, it remains possible that unmeasured confounding factors could introduce bias. The precision of our estimates could be influenced by the sample size as well. Moreover, as with any genetic study, population stratification could be a source of bias.

## 5 Conclusion

Our MR analysis provides insights into the potential relationships between serum lipid parameters and diabetic nephropathy. The results revealed a potential protective effect of high HDL-C levels as well as a possible adverse impact of elevated TG levels. Our analysis offers intriguing insights into the potential etiology and treatment target of diabetic nephropathy.

## Data availability statement

The original contributions presented in the study are included in the article/**Supplementary Material**. Further inquiries can be directed to the corresponding authors.

## Author contributions

HL: Writing – original draft. XY: Formal analysis, Writing – review & editing. LW: Software, Writing – original draft. JnL: Formal analysis, Writing – review & editing. XL: Validation, Writing – review & editing. XF: Investigation, Writing – review & editing. JaL: Methodology, Writing – review & editing. SD: Supervision, Writing – review & editing. YW: Formal analysis, Supervision, Writing – review & editing.

## Supplementary material

Supplementary Figure 1 Sensitivity analysis using “Leave-One-Out” method.

Supplementary Figure 2 Forest plot.

Supplementary Figure 3 Scatter plot.
