# Supplementary figures and images for: The causal relationship between 5 serum lipid parameters and diabetic nephropathy: a Mendelian randomization study
Source: Front Endocrinol (Lausanne). 2024 May 28;15:1358358. doi: 10.3389/fendo.2024.1358358 (PMC11165179; doi:10.3389/fendo.2024.1358358)

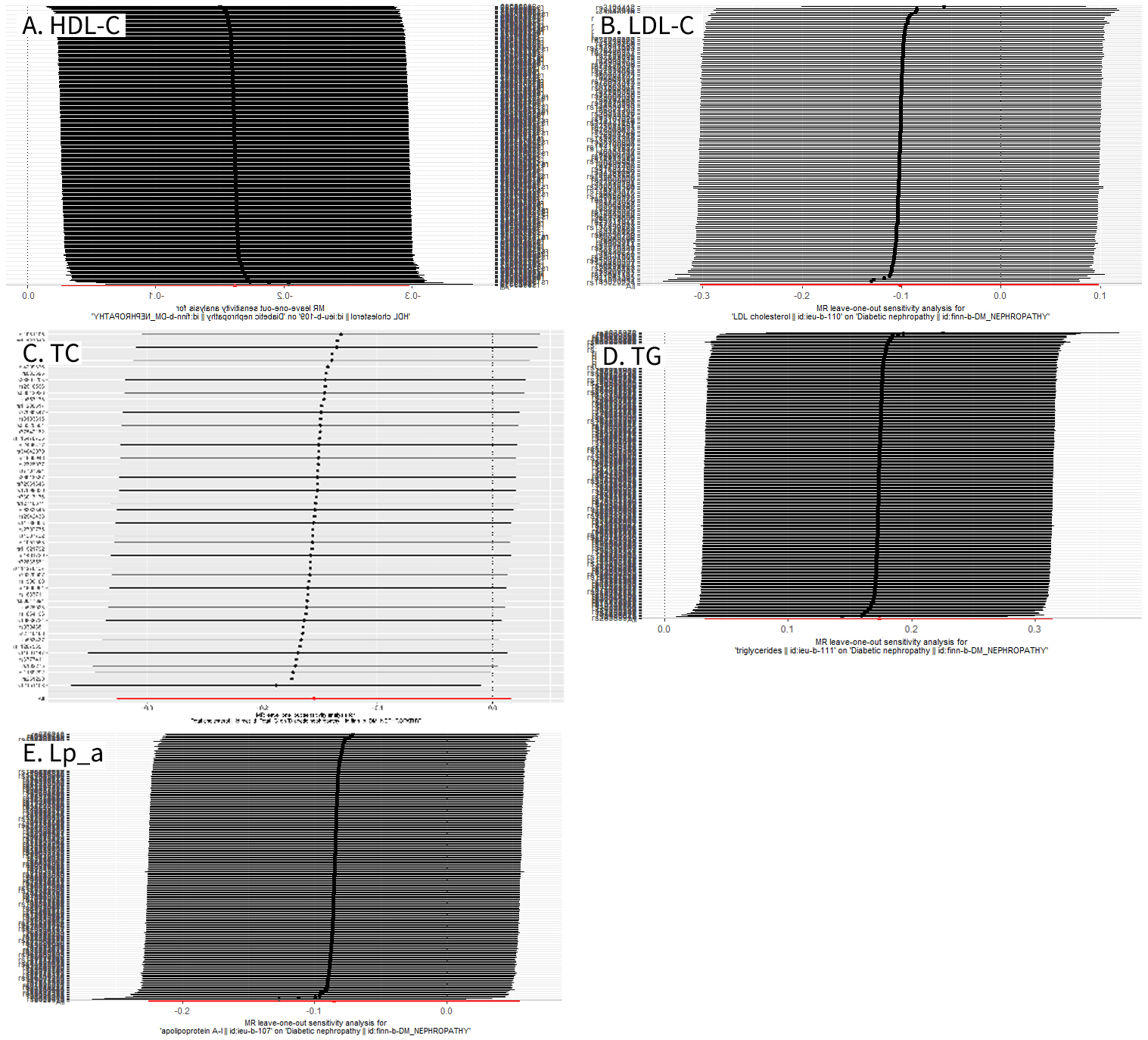

Supplement: Supplementary Figure 1 — Sensitivity analysis using “Leave-One-Out” method. [file Image_1.jpeg]

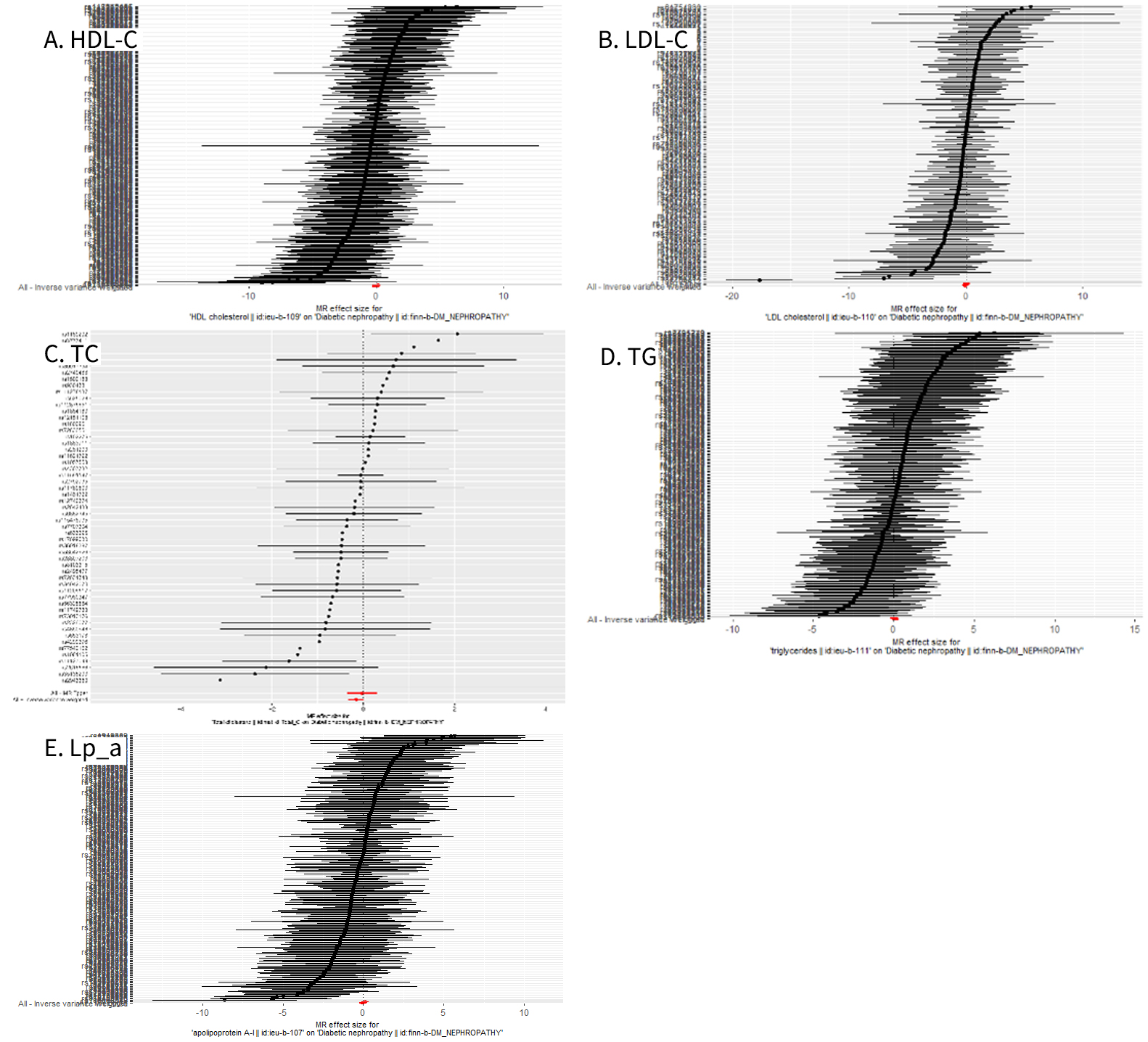

Supplement: Supplementary Figure 2 — Forest plot. [file Image_2.jpeg]

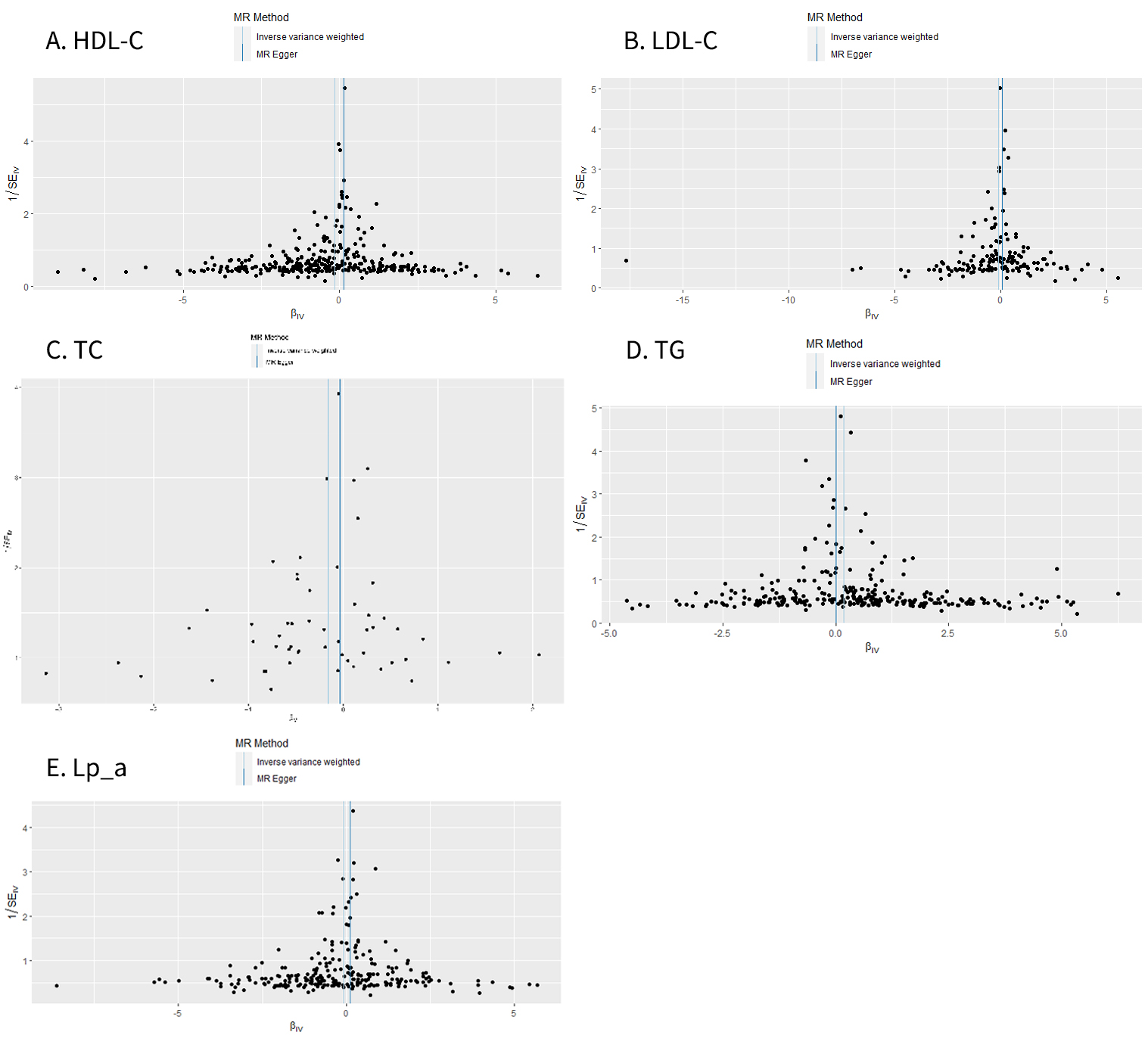

Supplement: Supplementary Figure 3 — Scatter plot. [file Image_3.jpeg]
